# Supplementary material for: Geomagnetic spikes on the core-mantle boundary
Source: Nat Commun. 2017 May 30;8:15593. doi: 10.1038/ncomms15593 (PMC5459996; doi:10.1038/ncomms15593)
Supplement: Supplementary Information — Supplementary Figures, Supplementary Table and Supplementary References [file ncomms15593-s1.pdf]

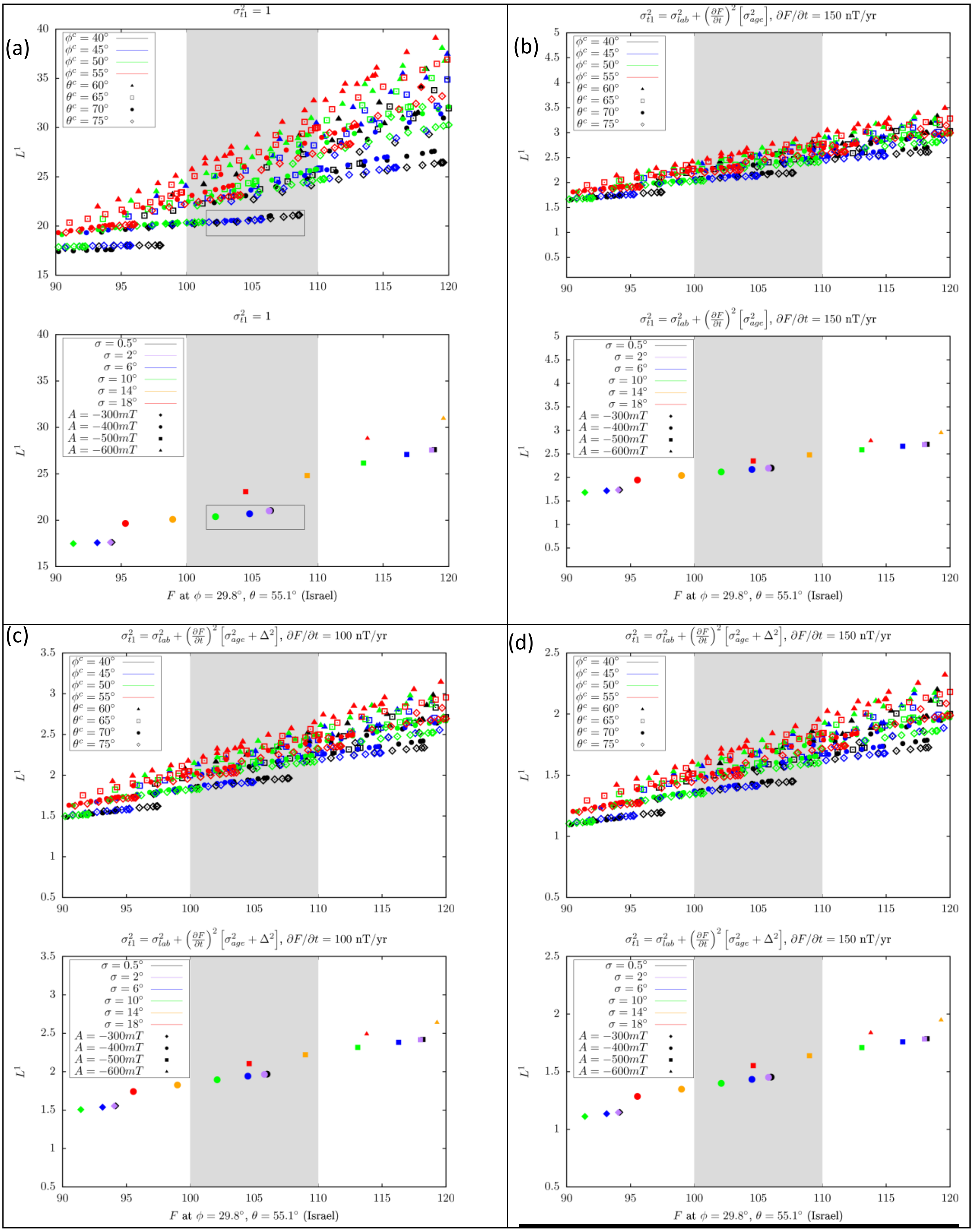

Supplementary Figure 1. Misfit to the 144 Levantine spike data using different measures of data misfit. Each panel contains two representations of the same 750 models with different parameter dependencies highlighted. The panels show different treatments of data uncertainties as described in the text: (a) shows all data weighted equally; (b) uses age and laboratory uncertainties but no age bias; (c) and (d) show the effect of varying $\partial F/\partial t$ when all three sources of uncertainty are included. The grey shaded region marks the spike intensity in Israel[1] with generous error bars applied. Top row: highlights dependence on location, where ϕ^c is indicated by colours and θ^c is shown by shape. Bottom row: CMB spike location is fixed at $\theta^c = 70°$ and $\phi^c = 40°$ to illustrate dependence on A and σ.

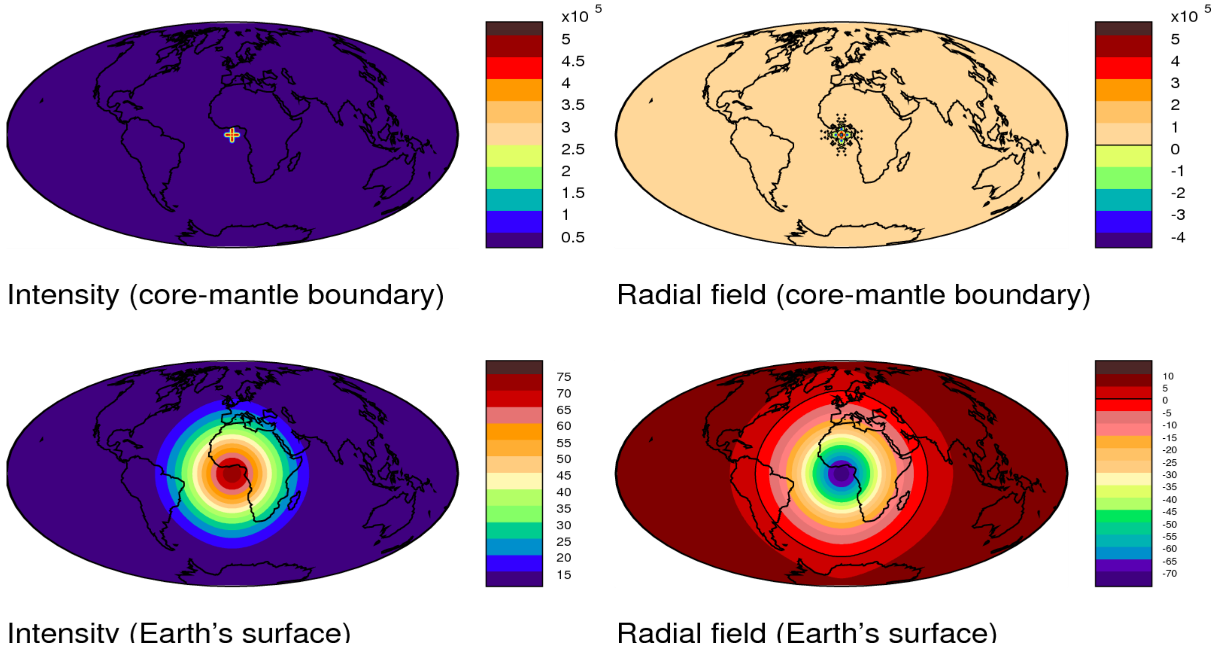

Supplementary Figure 2. Intensity F (left) and radial magnetic field B_r (right) at the core-mantle boundary (top) and Earth's surface (bottom) for nine spikes separated uniformly by $\Delta = 2°$. The other parameters are $A = 500mT$ and $\sigma = 1°$, $\theta^c = 90°$ and $\phi^c = 0°$. Colour scale is in μT.

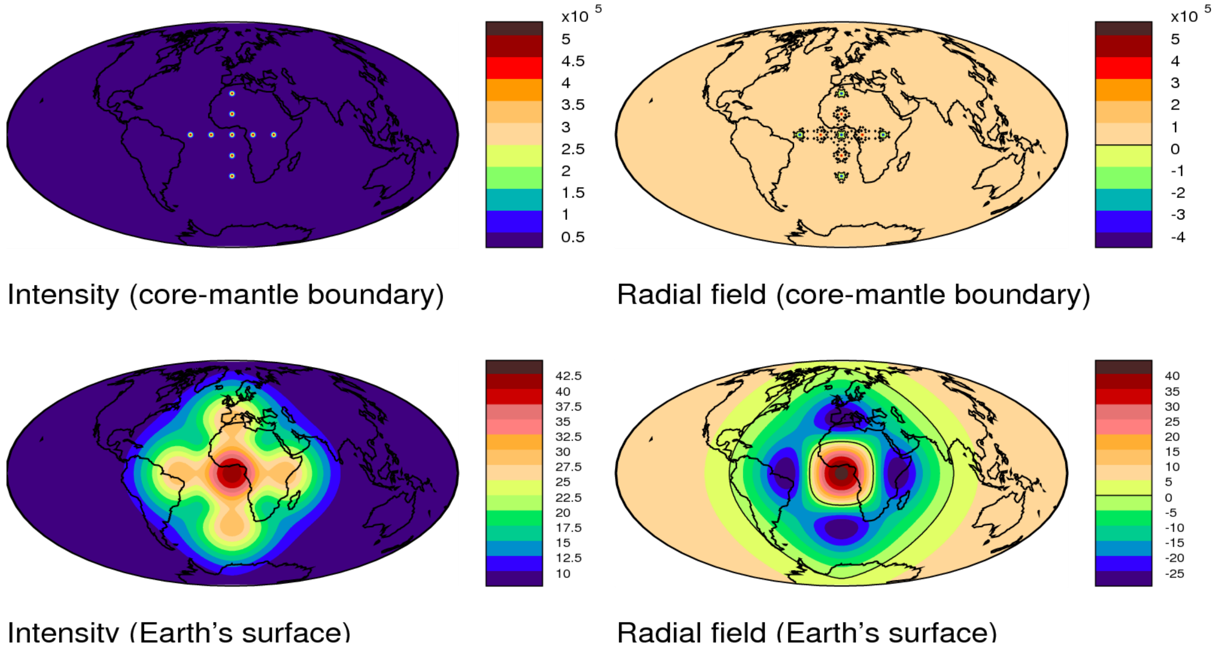

Supplementary Figure 3. Intensity F (left) and radial magnetic field B_r (right) at the core-mantle boundary (top) and Earth's surface (bottom) for nine spikes separated uniformly by $\Delta = 15°$. The other parameters are $A = 500mT$ and $\sigma = 1°$, $\theta^c = 90°$ and $\phi^c = 0°$. Colour scale is in μT.

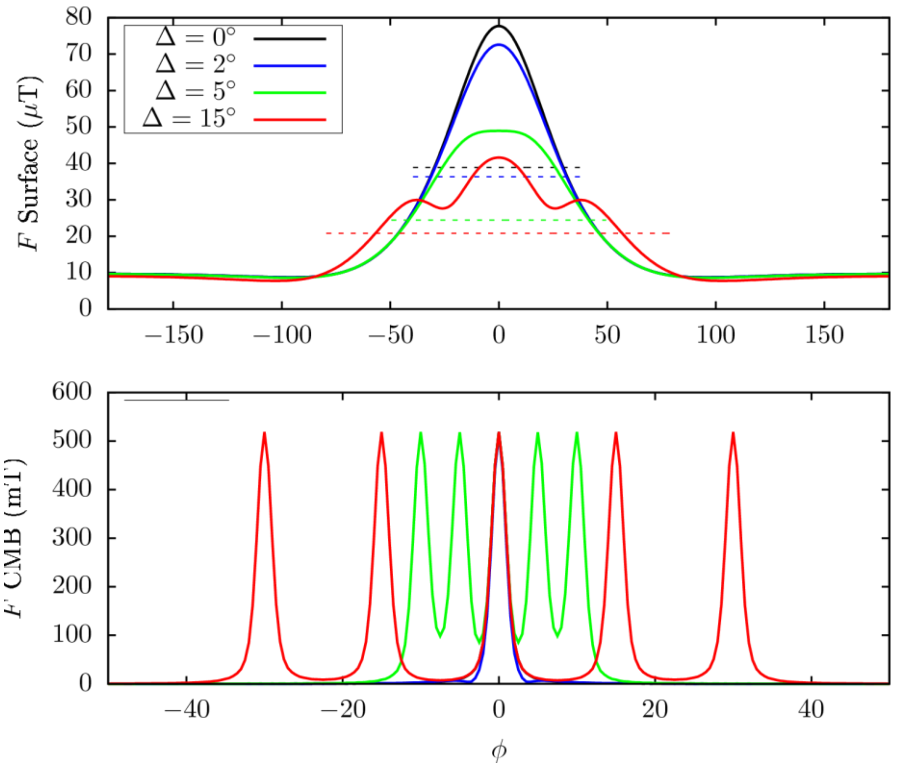

Supplementary Figure 4. Intensity F as a function of longitude ϕ at the surface (top) and the core-mantle boundary (bottom) for arrays of nine spikes. The other parameters are $A = 500mT$ and $\sigma = 1°$, $\theta^c = 90°$ and $\phi^c = 0°$. Note the different scale for the ordinate in the two plots. Dashed horizontal lines in the top plot show the width at half maximum (δ_2 in the main text).

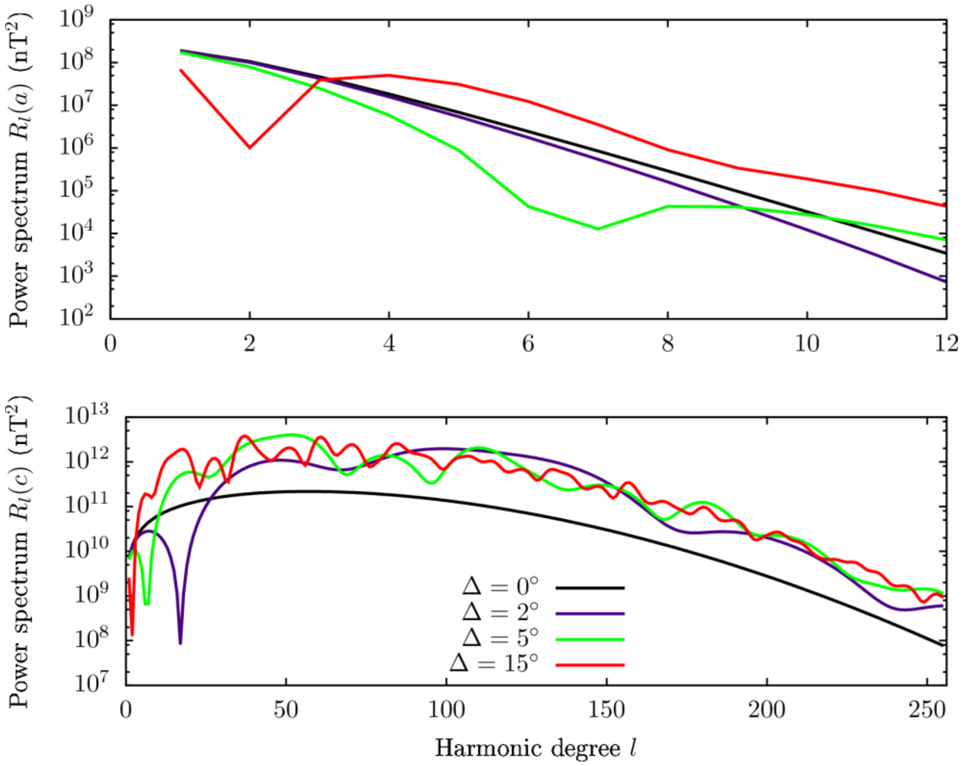

Supplementary Figure 5. Power spectrum R as a function of spherical harmonic degree l at the surface (top) and core-mantle boundary (bottom) for the spikes shown Supplementary Figures 2 and 3. Note the different scale for the ordinate in the two plots.

Location	Age	λ	ϕ	F	σ_{lab}	σ_{age}	Δ	$\sigma_{t1}(0.1)$	$\sigma_{t1}(0.15)$	$\sigma_{t1}(0.2)$	$\sigma_t(0.1)$	$\sigma_t(0.15)$
Mali	-865	15.4	354.5	42.7	5	25	135	5.59	6.25	7.07	14.61	21.19
Hawaii	-890	19.5	204.2	89.6	5	60	110	7.81	10.430	13	13.49	19.45
India	-954	25.5	77.0	48	5.9	99	46	11.52	15.98	20.66	12.41	17.40
Egypt	-1130	25.7	32.6	63	5	110	-130	12.08	17.24	22.56	17.75	26.03
Israel	-980	29.8	34.9	104.2	6.3	110	20	12.68	17.66	22.88	16.78	23.17
Jordan	-999	30.7	35.4	129.7	16.3	83	1	18.29	20.51	23.26	20.51	23.50
Japan	-1050	34.7	139.4	66.1	5	1500	-50	150.08	225.06	300.04	150.17	225.18
Cyprus	-1025	34.7	33.8	70.3	5	25	-25	5.59	6.25	7.07	6.12	7.29
Syria	-1150	34.9	40.6	59.2	5	50	-150	7.07	9.014	11.18	16.58	24.23
Iraq	-1095	36.0	43.3	66.7	5	19	-95	5.35	5.76	6.28	10.90	15.37
Greece	-875	38.0	23.7	57	5	25	125	5.59	6.25	7.07	13.69	19.76
Turkmen'n	-850	38.0	62.0	76.6	7.7	50	150	9.18	10.75	12.62	17.59	24.94
Uzbekistan	-940	38.1	67.8	77.2	7.7	20	60	7.96	8.26	8.68	9.96	12.22
Turkey	-1050	38.4	38.4	100.8	7	150	-50	16.55	23.56	30.81	17.29	24.73
Portugal	-880	38.5	7.3	85.4	5	110	120	12.08	17.24	22.56	17.03	24.93
Georgia	-1150	41.7	46.1	99	12.1	50	-150	13.09	14.24	15.70	19.91	26.63
Bulgaria	-1050	42.2	24.8	77.8	5	50	-50	7.07	9.014	11.18	8.66	11.73
Serbia	-900	45.2	20.1	70.7	6.7	100	100	12.04	16.43	21.09	15.65	22.25
Switzerland	-1025	46.6	6.5	50.3	5	105	-25	11.63	16.52	21.59	11.90	16.94
Moldova	-1150	47.0	29.0	75	5	50	-150	7.07	9.01	11.18	16.58	24.24
Ukraine	-850	50.2	30.7	83	5	50	150	7.07	9.01	11.18	16.58	24.24
Czech	-1150	50.4	13.9	62.2	6.2	250	-150	25.76	38.01	50.38	29.80	44.17
China	-1000	53.0	107.0	93.5	9.3	200	0	22.06	31.41	41.07	22.06	31.41

Supplementary Table 1. Summary of data corresponding to the peak paleointensity measurement obtained at each of the 23 locations during the interval 1150 – 850 BC. Age in BC, latitude λ and longitude ϕ in degrees, intensity F in μT, intensity uncertainty σ_{lab} in μT, age uncertainty σ_{age} and age bias Δ in yrs. σ_{t1} and σ_t are the total uncertainty excluding and including age bias respectively. Numbers in brackets in the heading for the last four columns indicate the value of $\partial F/\partial t$ (μT) used to construct the estimate.

Supplementary References

1. Shaar, R. *et al.* Geomagnetic field intensity: How high can it get? How fast can it change? Constraints from Iron Age copper slag. *Earth Planet. Sci. Lett.* **301,** 297–306 (2011).
